# A Single Polyoxometalate Nanozyme for Cross-Reactive Thiol Array Sensing via pKa-Driven and Enrichment-Synergistic Strategy

**DOI:** 10.3390/s26103175

**Published:** 2026-05-17

**Authors:** Baohong Sun, Ying Liu, Xinxin Tian, Yu Fang, Jinpei Mei, Yang Chen, Tao Ma

**Affiliations:** Anhui Engineering Technology Research Center of Biochemical Pharmaceutical, School of Pharmacy, Bengbu Medical University, Bengbu 233030, China

**Keywords:** nanozyme, array sensor, biothiols, pH regulation, disease diagnosis

## Abstract

**Highlights:**

**What are the main findings?**

- A single-material three-channel colorimetric array sensor based on Fe_4_P_2_W_18_ polyoxometalate nanozyme achieves 100% discrimination accuracy for glutathione, cysteine, and homocysteine within 5 min.
- By exploiting pH-dependent stepped catalytic responses (pH 3.5, 4.0, 4.5), the sensor amplifies pKa-driven differences in biothiol molecular charge and structure, enabling a detection limit as low as 0.1 μM and quantification over 1–50 μM.

**What are the implications of the main findings?**

- This strategy eliminates the need for multi-material sensor arrays while providing mechanistic clarity, offering a simple, rapid, and reliable tool for biothiol analysis in complex biological samples.
- The sensor successfully distinguishes different cell types (HUVEC, HeLa, A549) and discriminates serum samples from cardiovascular disease patients, demonstrating strong potential for early disease diagnosis.

**Abstract:**

Glutathione (GSH), cysteine (Cys), and homocysteine (Hcy) are essential biothiols involved in redox homeostasis and cell signaling. Yet, their structural similarity poses a significant challenge for accurate discrimination in complex biological samples. Herein, we report a polyoxometalate nanozyme (Fe_4_P_2_W_18_) with an atomically precise structure synthesized via a mild one-step method. Benefiting from its negatively charged surface and multi-electron reduction centers, the nanozyme effectively enriches biothiols under acidic conditions and exhibits excellent peroxidase-like activity. By leveraging its stepped catalytic response across pH 3.5, 4.0, and 4.5, which amplifies the pKa-driven differences in the overall molecular charge and electrostatic properties of biothiols, we constructed a single-material three-channel colorimetric array sensor that generates cross-reactive fingerprints. This sensor achieves 100% discrimination accuracy for GSH, Cys, and Hcy within 5 min, with a detection limit of 0.1 μM. It enables quantitative detection of thiol concentrations between 1 and 50 μM and accurately identifies binary and ternary thiol mixtures. Furthermore, the sensor successfully distinguishes cell types (HUVEC, HeLa, A549) and discriminates serum samples from cardiovascular disease patients. This strategy eliminates the complexity of multi-material arrays while leveraging the well-defined structure of Fe_4_P_2_W_18_ to provide mechanistic clarity, offering a simple, rapid, and reliable tool for biothiol analysis and early disease diagnosis.

## 1. Introduction

GSH, Cys, and Hcy are key low-molecular-weight biothiols involved in redox homeostasis, protein modification, and cell signaling [1,2]. Their abnormal levels are linked to various diseases: In human serum, normal physiological concentrations of GSH and Hcy typically range from 1 to 15 μM and 5 to 15 μM, respectively, while Cys is present at higher levels (~200–300 μM). Elevated Hcy (>15 μM) is an established independent risk factor for cardiovascular disease, and depleted GSH levels are associated with cancer and neurodegenerative disorders [3,4]. Despite their physiological significance, these thiols share high structural similarity, differing only in carbon chain length and side chains, making their accurate discrimination in complex samples challenging [5,6]. Traditional methods such as high-performance liquid chromatography and mass spectrometry provide sensitive quantification but require expensive equipment and labor-intensive pretreatment, thereby limiting point-of-care use [7,8]. Thus, simple, rapid sensing strategies enabling effective discrimination of structurally similar thiols are of great value for disease diagnosis.

Colorimetric nanozyme sensors have garnered widespread attention in biothiol detection in recent years due to their advantages, including simple operation, rapid response, and visual readout [9,10,11]. However, conventional nanozyme sensors generally follow a “lock-and-key” recognition mode, in which one sensing element selectively responds to a single analyte [12]. Owing to the high structural similarity among GSH, Cys, and Hcy, effective discrimination among the three using a single nanozyme sensing unit remains challenging [13]. To overcome this limitation, nanozyme-based array sensors can generate unique response “fingerprints” through an inhibition mechanism [14]. For instance, Zheng et al. developed a nanozyme array based on two types of metal ion-doped carbon dots, which improved the detection sensitivity for biothiols [15]. However, most reported array sensors for biothiol detection are based on strategies such as multi-material combinations, synergistic integration of single materials with environmental regulation, and confinement/microenvironment enhancement [16,17,18,19], with few strategies focusing on the synergistic regulation of nanozyme catalysis and thiol protonation states.

Among nanozyme materials, polyoxometalates (POMs) have recently emerged as promising sensing platforms owing to their unique combination of properties. Unlike many conventional nanozymes whose active sites and surface chemistry are poorly defined, POMs possess atomically precise molecular structures, well-characterized multi-electron redox centers, and inherently high negative charge density. These features confer several advantages for biothiol sensing: (i) the structural precision facilitates mechanistic understanding of catalytic and recognition processes; (ii) the highly negatively charged surface enables electrostatic enrichment of positively charged target molecules, enhancing sensitivity; and (iii) the peroxidase-like activity can be modulated by pH and composition. Despite these advantages, the use of a single POM nanozyme to construct a cross-reactive sensor array for discriminating structurally similar biothiols has not been reported.

The thiol (-SH) group of biothiols (GSH, Cys, Hcy) is weakly acidic and exists in a protonation-deprotonation equilibrium [20]. Under neutral or alkaline conditions, the proportion of thiolate (-S^−^) is high, leading to significantly enhanced nucleophilicity and stronger interactions with nanozymes, thereby resulting in a more pronounced inhibition effect [21,22]. In contrast, under acidic conditions, although the nucleophilicity (reducing ability) of the thiol group is diminished, making detection more challenging, the differences in protonation states among the three thiols become more pronounced due to their distinct pKa values [23]. These differences are further amplified across three pH gradients, generating highly distinctive “fingerprint responses” [24]. At these acidic pH values, the thiol groups remain predominantly protonated; the discriminatory power arises instead from the distinct net molecular charges and sizes governed by the protonation states of the α-amino, α-carboxyl, and side-chain groups. This necessitates that the nanozyme employed for array construction possesses a specific structure-activity relationship toward biothiols to enhance sensitivity under acidic conditions.

**Scheme 1:**
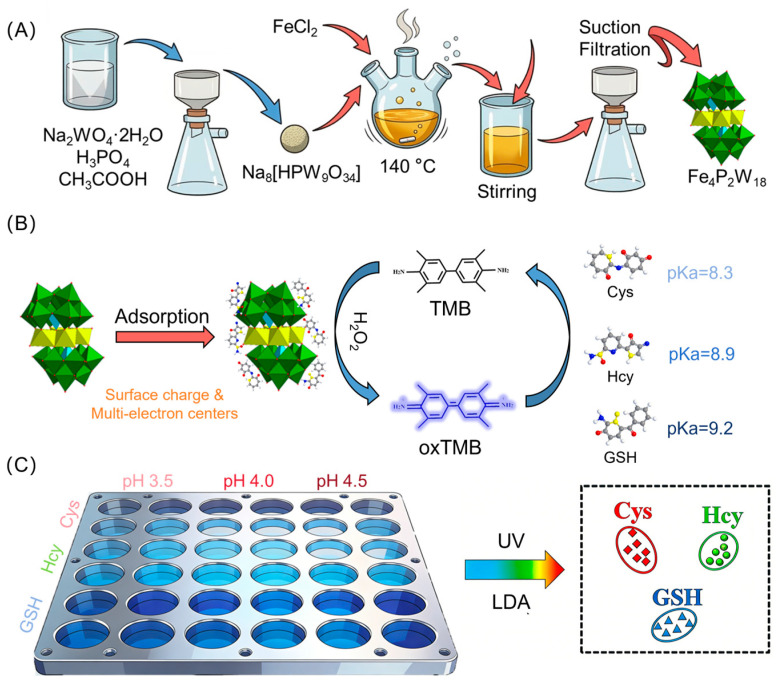
(**A**) Schematic illustration of the synthesis of Fe_4_P_2_W_18_ nanozyme. (**B**) Fe_4_P_2_W_18_ nanozyme for biothiol determination via a pKa-driven and enrichment-synergistic strategy. (**C**) Construction of a biothiol sensing array based on the Fe_4_P_2_W_18_ nanozyme for the analysis of GSH, Cys, and Hcy.

Herein, we synthesized a polyoxometalate nanozyme (Fe_4_P_2_W_18_) with an atomically precise structure via a mild one-step method (Scheme 1A). Due to the anion clusters and multi-electron reduction centers of the polyoxometalate framework, it can enrich biothiols through surface negative charges and electron transfer, thereby enhancing the specific detection capability for biothiols under acidic conditions (Scheme 1B). Leveraging the stepped catalytic response of this nanozyme under different pH conditions, we constructed a three-channel colorimetric array sensor using a single material. We employed three sensing units at pH 3.5, 4.0, and 4.5 to generate cross-reactive fingerprints, achieving 100% discrimination accuracy for GSH, Cys, and Hcy within 5 min. The resulting three-channel response vectors were processed by Linear Discriminant Analysis (LDA), a supervised pattern recognition algorithm that maximizes inter-class separation while minimizing intra-class variance, and Hierarchical Cluster Analysis (HCA), which provides complementary unsupervised grouping evidence (Scheme 1C). The response speed outperforms most previously reported colorimetric array sensors. This strategy eliminates the cumbersome steps associated with multi-material synthesis and formulation required by conventional array sensors. Meanwhile, the well-defined structure of Fe_4_P_2_W_18_ provides a reliable foundation for elucidating the catalytic and sensing mechanisms. Furthermore, this sensor was successfully applied to identify cell types (HUVEC, HeLa, A549) and to discriminate serum samples from patients with cardiovascular disease, demonstrating its promising potential for early disease diagnosis.

## 2. Materials and Methods

### 2.1. Chemical and Materials

Sodium tungstate dihydrate (Na_2_WO_4_·2H_2_O, ≥99%), phosphoric acid (H_3_PO_4_, 85%), acetic acid (CH_3_COOH, ≥99.5%), ferrous chloride (FeCl_2_, ≥98%), potassium chloride (KCl, ≥99.5%), and hydrogen peroxide (H_2_O_2_, 30%) were purchased from Sinopharm Chemical Reagent Co., Ltd. (Shanghai, China). 3,3′,5,5′-Tetramethylbenzidine (TMB, ≥99%) was obtained from Aladdin Industrial Corporation (Shanghai, China). Glutathione (GSH, ≥98%), cysteine (Cys, ≥98%), and homocysteine (Hcy, ≥95%) were purchased from Sigma-Aldrich (St. Louis, MO, USA). Terephthalic acid (TA, ≥99%) was used as a fluorescence probe for hydroxyl radical (·OH) detection. All reagents were of analytical grade and used without further purification. Deionized water (18.2 MΩ·cm) was used throughout all experiments.

### 2.2. Characterization

The morphology of the Fe_4_P_2_W_18_ nanozyme was characterized by scanning electron microscopy (SEM, JSM-7800F, JEOL, Tokyo, Japan). X-ray diffraction (XRD) patterns were recorded on a D8 Advance diffractometer (Bruker, Karlsruhe, Germany) using Cu Kα radiation (λ = 1.5418 Å). Zeta potential measurements were performed on a Zetasizer Nano ZS90 (Malvern Instruments, Malvern, UK). Fourier-transform infrared (FT-IR) spectra were collected on a Nicolet iS10 spectrometer (Thermo Fisher Scientific, Waltham, MA, USA) using KBr pellets. X-ray photoelectron spectroscopy (XPS) analysis was conducted on an ESCALAB 250Xi spectrometer (Thermo Fisher Scientific, Waltham, MA, USA) with Al Kα radiation to determine elemental composition and chemical states. Energy-dispersive X-ray spectroscopy (EDS) was employed to verify the elemental distribution.

### 2.3. Synthesis of Na_8_[HPW_9_O_34_]·24H_2_O

The precursor Na_8_[HPW_9_O_34_]·24H_2_O was synthesized according to a previously reported method with slight modifications [25]. Typically, 120 g of Na_2_WO_4_·2H_2_O was dissolved in 150 mL of H_2_O at 80 °C under vigorous stirring. Subsequently, 3 mL of 85% H_3_PO_4_ was added, followed by the dropwise addition of 22 mL of CH_3_COOH within 30 min. After the formation of a white precipitate, the mixture was filtered. The precipitate was washed with 4 M NaCl solution, collected by filtration, and dried at 60 °C to obtain the final product.

### 2.4. Synthesis of Fe_4_P_2_W_18_ Nanozyme

The Fe_4_P_2_W_18_ polyoxometalate nanozyme was prepared via a mild one-step method. In a typical procedure, FeCl_2_ was dissolved in 40 mL of deionized water, and the pH was adjusted to 6.0. Na_8_[HPW_9_O_34_]·24H_2_O was then added at a molar ratio of FeCl_2_ to Na_8_[HPW_9_O_34_]·24H_2_O of 2.5:1, based on preliminary optimization of catalytic activity. The mixture was refluxed at 140 °C for 90 min. After cooling to room temperature, 7 g of KCl was added and stirred for 20 min. The resulting precipitate was collected by vacuum filtration, washed three times with deionized water to remove residual KCl and soluble impurities, and finally dried under vacuum at 60 °C until constant weight.

### 2.5. Peroxidase-like Activity Assay

The peroxidase-like activity of Fe_4_P_2_W_18_ was evaluated using the classic TMB chromogenic reaction. Typically, Fe_4_P_2_W_18_ (12 μg/mL), TMB (2 mM), and H_2_O_2_ (1 mM) were mixed in acetate buffer (pH 3.5) and incubated at 45 °C for 5 min. The absorbance of the reaction mixture was recorded at 652 nm using a UV-vis spectrophotometer (UV-2600, Shimadzu, Japan). Steady-state kinetic studies were performed by varying the concentration of TMB or H_2_O_2_ while keeping the other substrate constant. The kinetic parameters (Km and Vmax) were calculated using the Lineweaver-Burk double-reciprocal plot.

### 2.6. Detection Mechanism Investigation

The generation of hydroxyl radicals (·OH) during the catalytic process was confirmed using terephthalic acid (TA) as a fluorescent probe and electron paramagnetic resonance (EPR) spectroscopy. For the TA assay, Fe_4_P_2_W_18_, TA, and H_2_O_2_ were incubated in acetate buffer, and the fluorescence emission intensity was measured at 425 nm with excitation at 315 nm. EPR spectra were recorded on a Bruker EMXplus spectrometer (Bruker, Billerica, MA, USA) using 5,5-dimethyl-1-pyrroline N-oxide (DMPO) as the spin-trapping agent.

### 2.7. Colorimetric Array Sensor Construction and Thiol Discrimination

A single-material three-channel array sensor was constructed based on the pH-dependent catalytic activity of Fe_4_P_2_W_18_. The detection was performed in acetate buffer at three different pH values (3.5, 4.0, and 4.5). For each channel, Fe_4_P_2_W_18_ (12 μg/mL), TMB (2 mM), H_2_O_2_ (1 mM), and varying concentrations of biothiols (GSH, Cys, or Hcy) were mixed and incubated at 45 °C for 5 min. The absorbance at 652 nm was recorded. The signal response (ΔA) was calculated as ΔA = A_0_ − A, where A_0_ and A represent the absorbance in the absence and presence of biothiols, respectively. The three-channel response patterns were analyzed by linear discriminant analysis (LDA) and hierarchical cluster analysis (HCA) using SPSS Statistics version 26.0 (IBM, Armonk, NY, USA).

### 2.8. Analytical Performance Evaluation

The stability, reproducibility, repeatability, and selectivity of the Fe_4_P_2_W_18_ sensor array were systematically evaluated. For stability, the nanozyme was stored at room temperature, and its catalytic activity was measured daily over 7 days using the standard TMB assay (Fe_4_P_2_W_18_: 12 μg/mL, TMB: 2 mM, H_2_O_2_: 1 mM, acetate buffer pH 3.5, 45 °C, 5 min). Reproducibility was assessed by synthesizing five independent batches of Fe_4_P_2_W_18_ nanozyme, and each batch was tested for the detection of GSH, Cys, and Hcy at 10 μM under the optimized conditions; the RSD of the ΔA values across the five batches was calculated for each analyte. Repeatability was determined by performing ten successive measurements of GSH (10 μM) using a single sensor, and the RSD was calculated. For selectivity, a mixed interferent solution containing glucose, glycine, lysine, ascorbic acid, Na^+^, and K^+^ (100 μM each) and BSA (100 μg/mL) was prepared in acetate buffer. The signal response (ΔA) of GSH, Cys, and Hcy (10 μM each) was first measured individually, and then each analyte was spiked into the interferent mixture and measured again. The recovery was calculated as (ΔA_in mixture_/ΔA_alone_) × 100%. All experiments were performed in triplicate.

### 2.9. Real Sample Analysis

All procedures involving human samples were approved by the Medical Research Ethics Committee of Bengbu Medical University (approval No. 2025476) and were performed in accordance with the principles of the Declaration of Helsinki. Written informed consent was obtained from all participants and donors. Human serum samples were collected from healthy volunteers and patients with cardiovascular disease. Cell lines, including human umbilical vein endothelial cells (HUVEC), HeLa cells, and A549 cells, were cultured and processed to obtain cell lysates. The prepared sensor array was applied to these real samples following the same protocol described above. Each experiment was performed in triplicate to ensure reproducibility. Human serum and HeLa cell lysate were diluted 10-fold with acetate buffer (0.1 M, pH 3.5). Each sample was spiked with GSH, Cys, or Hcy at a final added concentration of 10 μM. The three-channel sensor array was applied under optimized conditions (Fe_4_P_2_W_18_: 12 μg/mL, TMB: 2 mM, H_2_O_2_: 1 mM, pH 3.5/4.0/4.5, 45 °C, 5 min), and the ΔA response was recorded. Measured concentrations were derived from the LDA1 calibration curves. Recovery was calculated as (C_measured_ − C_unspiked_)/C_spiked_ × 100%.

## 3. Results and Discussion

### 3.1. Synthesis and Characterization of Fe_4_P_2_W_18_ Nanozyme

**Figure 1:**
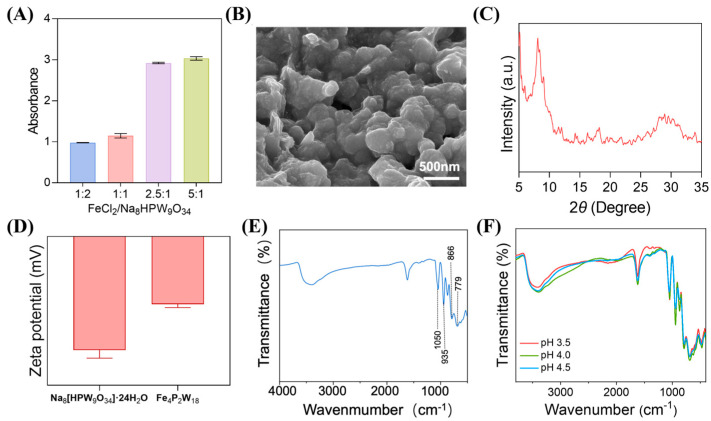
(**A**) Effect of different feeding ratios on the catalytic performance of Fe_4_P_2_W_18_. (**B**) SEM image of Fe_4_P_2_W_18_ nanozymes. (**C**) XRD pattern of Fe_4_P_2_W_18_ nanozymes. (**D**) Zeta potential spectrum of Na_8_[HPW_9_O_34_]·24H_2_O and Fe_4_P_2_W_18_ nanozymes. (**E**) FT-IR spectrum of Fe_4_P_2_W_18_ nanozymes. (**F**) FT-IR spectra of Fe_4_P_2_W_18_ nanozymes under different pH values for 6 h of culturing.

The Fe_4_P_2_W_18_ polyoxometalate nanozyme was synthesized via a mild one-step method using Na_8_[HPW_9_O_34_]·24H_2_O and FeCl_2_ as precursors. To obtain a nanozyme with high catalytic activity, the molar ratio of FeCl_2_ to Na_8_[HPW_9_O_34_] was first optimized. As shown in Figure 1A, when the ratio reached 2.5:1, the absorbance at 652 nm increased rapidly, while further increasing the FeCl_2_ content resulted in negligible improvement. Considering both catalytic efficiency and economic cost, a ratio of 2.5:1 was selected for subsequent experiments. The morphology and composition of Fe_4_P_2_W_18_ were characterized by SEM, XRD, Zeta potential analysis, and FT-IR spectroscopy. The SEM image (Figure 1B) reveals that the Fe_4_P_2_W_18_ is composed of numerous small nanocrystals with particle sizes in the range of several hundred nanometers. The XRD pattern of Fe_4_P_2_W_18_ (Figure 1C) exhibits a distinct sharp peak at approximately 2*θ* = 8°, indicative of a well-defined periodic arrangement of the polyoxometalate clusters, which is consistent with its atomically precise structure [26]. Meanwhile, a broad hump in the 2*θ* range of 20–35° is observed, suggesting the nanocrystalline nature of the material [27]. This combined feature is typical for nanoscale polyoxometalate aggregates, where the primary clusters maintain long-range order along specific crystallographic directions. Meanwhile, the overall particle size and surface disorder lead to peak broadening at higher diffraction angles [28]. This unique structural characteristic provides both high catalytic activity due to the exposed active sites and structural stability from the cluster framework. The precursor Na_8_[HPW_9_O_34_] exhibits a highly negative Zeta potential (approximately −30 mV), confirming the strong electrostatic driving force that is retained in the final Fe_4_P_2_W_18_ nanozyme (Figure 1D, −18 mV). This negatively charged surface enhances the affinity toward positively charged substrates, thereby improving its peroxidase-like catalytic performance. FT-IR spectroscopy (Figure 1E) further verified the chemical composition, with characteristic peaks at 779, 866, 935, and 1050 cm^−1^ corresponding to W–O_c_–W, W–O_b_–W, W_1/4_O_d_, and P–O_a_ bonds, respectively [25]. The presence of these characteristic peaks, together with the negatively charged surface revealed by Zeta potential, confirms the structural integrity of Fe_4_P_2_W_18_ and provides the necessary chemical basis for its enrichment of biothiols. The hydrolytic stability of the Fe_4_P_2_W_18_ framework across the working pH range was confirmed by FT-IR spectra recorded after incubation at pH 3.5, 4.0, and 4.5, which showed no changes in the characteristic POM vibrational bands (Figure 1F).

**Figure 2:**
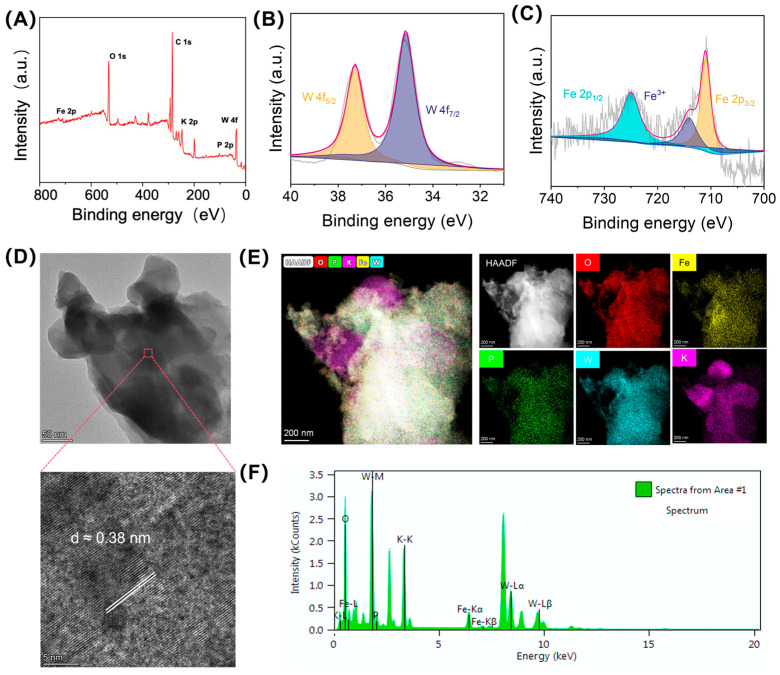
(**A**) XPS survey spectrum of Fe_4_P_2_W_18_ nanozymes. (**B**) High-resolution W 4f spectrum of the Fe_4_P_2_W_18_. (**C**) High-resolution Fe 2p spectrum of the Fe_4_P_2_W_18_. (**D**) HRTEM images of Fe_4_P_2_W_18_ nanozymes. (**E**) EDS mapping of Fe_4_P_2_W_18_ nanozymes. (**F**) EDS spectrum of Fe_4_P_2_W_18_ nanozymes in STEM HAADF mode.

**Figure 3:**
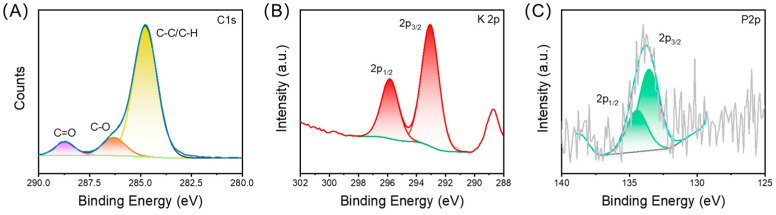
(**A**) High-resolution C 1s spectrum of the Fe_4_P_2_W_18._ (**B**) High-resolution K 2p spectrum of the Fe_4_P_2_W_18_. (**C**) High-resolution P 2p spectrum of the Fe_4_P_2_W_18_.

XPS analysis was employed to precisely determine the elemental composition and valence states of Fe_4_P_2_W_18_ (Figure 2A). The survey spectrum revealed characteristic peaks corresponding to P 2p, W 4f, Fe 2p, K 2p, and O 1s, which are in good agreement with the theoretical composition of the target compound. Quantitative atomic percentages were derived from the high-resolution narrow-scan analyses (Table 1). High-resolution XPS spectra were further acquired to elucidate the chemical states of tungsten and iron. The W 4f spectrum (Figure 2B) exhibits two distinct peaks at binding energies of approximately 35.6 eV and 37.7 eV, assigned to W 4f_7/2_ and W 4f_5/2_, respectively, with a spin–orbit splitting of 2.1 eV. These values are characteristic of W^6+^ in a typical polyoxometalate framework, confirming the fully oxidized state of tungsten. The Fe 2p spectrum (Figure 2C) shows two main peaks at approximately 711.2 eV and 724.8 eV, corresponding to Fe 2p_3/2_ and Fe 2p_1/2_, respectively, with a spin–orbit splitting of 13.6 eV (Figure 2C). Notably, the presence of a satellite peak at approximately 718.5 eV is a fingerprint of high-spin Fe^3+^ species, confirming that iron exists in the +3 oxidation state. These binding energy assignments are essential for the peroxidase-like catalytic activity of Fe_4_P_2_W_18_, as they confirm the retention of redox-active centers within the polyoxometalate structure [29]. The C 1s signal primarily serves as a binding energy reference for charge correction (Figure 3A). The K 2p doublet exhibits a characteristic spin–orbit splitting of approximately 2.8 eV, confirming the presence of K^+^ as the charge-balancing counterion within the polyoxometalate structure (Figure 3B). The P 2p signal, typically appearing as a single peak at around 133.5 eV for P^5+^, verifies the incorporation of phosphorus into the framework without the presence of reduced phosphorus species (Figure 3C). These features provide key evidence for the stability and structural integrity of the heteropoly Fe_4_P_2_W_18_ compound. The oxygen bonding environments, which would ideally be resolved by deconvolution of the O 1s XPS spectrum, were instead characterized by FT-IR spectroscopy (Figure 1E), where the distinct W–O_c_–W, W–O_b_–W, W=O_d_, and P–O_a_ vibrations provide unambiguous bond-level structural information complementary to the XPS data.

High-resolution transmission electron microscopy (HRTEM) was performed to further investigate the detailed microstructure of Fe_4_P_2_W_18_. As shown in Figure 2D, the nanozyme exhibits a distinct nanosheet-like morphology with lateral dimensions ranging from several hundred nanometers to approximately one micrometer. The nanosheets are composed of densely packed nanocrystalline domains with sizes of approximately 5–10 nm, consistent with the broad diffraction features observed in the XRD pattern. Notably, HRTEM imaging (Figure 2D) reveals well-resolved lattice fringes within these nanocrystalline regions, with an interplanar spacing of approximately 0.38 nm, corresponding to the characteristic spacing of the polyoxometalate framework. The coexistence of ordered lattice fringes and amorphous-like regions reflects the partially crystalline nature of the material, which is typical for nanoscale polyoxometalate aggregates. EDS mapping analysis (Figure 2E) further corroborated the structural homogeneity at the nanoscale, revealing the uniform distribution of W, O, Fe, and P elements, thereby demonstrating the successful construction of the W–O–Fe–P network within the polyoxometalate framework [30]. The corresponding energy-dispersive X-ray spectroscopy (EDS) spectrum exhibited characteristic X-ray peaks for W, O, Fe, and P, further confirming the elemental composition of the nanozyme (Figure 2F). Collectively, these characterizations confirm the successful synthesis of Fe_4_P_2_W_18_, with an atomically precise, well-defined structure.

### 3.2. POD-like Activity of Fe_4_P_2_W_18_ Nanozyme

**Figure 4:**
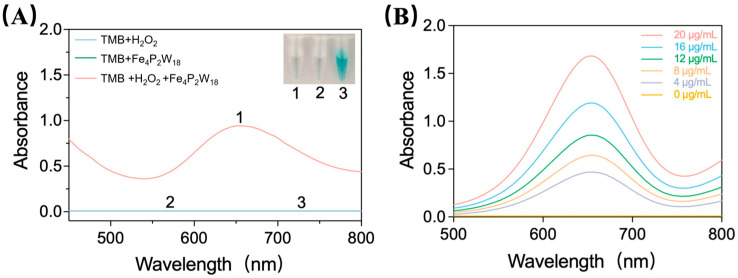
(**A**) UV-vis absorption spectra of TMB oxidation catalyzed by Fe_4_P_2_W_18_ nanozymes at 652 nm. (**B**) Effect of different concentrations of Fe_4_P_2_W_18_ nanozymes on catalytic activity.

The peroxidase-like activity of Fe_4_P_2_W_18_ was evaluated using the classic TMB chromogenic reaction. As shown in Figure 4A, when Fe_4_P_2_W_18_, TMB, and H_2_O_2_ were all present, a distinct absorption peak at 652 nm emerged, corresponding to the π–π* transition of oxidized TMB (oxTMB) [31]. In contrast, control experiments lacking any one of the three components (Fe_4_P_2_W_18_, TMB, or H_2_O_2_) exhibited negligible absorbance at 652 nm, confirming that the catalytic oxidation of TMB requires the simultaneous presence of the nanozyme and H_2_O_2_. Concurrently, the reaction solution turned from colorless to deep blue, providing a clear visual indication that Fe_4_P_2_W_18_ can utilize H_2_O_2_ as a substrate to generate highly reactive hydroxyl radicals (·OH) that oxidize TMB [32]. The effect of Fe_4_P_2_W_18_ concentration on catalytic activity was subsequently investigated. As illustrated in Figure 4B, the absorbance at 652 nm increased progressively with increasing nanozyme concentration in the range of 0–20 μg/mL, indicating a concentration-dependent catalytic response. A concentration of 12 μg/mL was selected as the optimal dosage for subsequent experiments, providing a suitable balance between high catalytic activity and economic efficiency.

**Figure 5:**
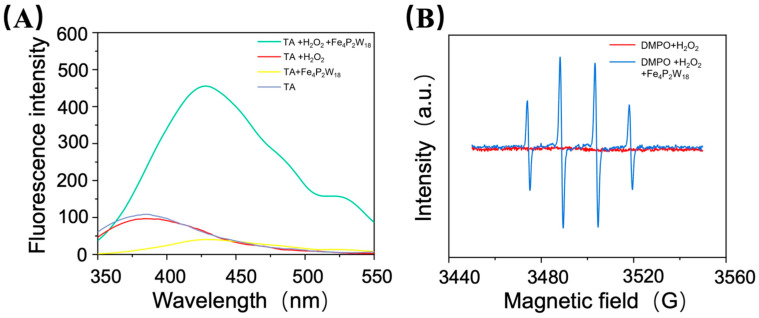
(**A**) Investigation of catalytic mechanism using TA as a fluorescent probe; (**B**) Electron paramagnetic resonance (EPR) spectrum confirming the generation of ·OH.

To clearly understand the catalytic process of H_2_O_2_ by the Fe_4_P_2_W_18_ nanomaterial, we conducted a series of experiments. First, we selected terephthalic acid (TA), a fluorescent probe with high specificity for hydroxyl radicals (·OH). As shown in Figure 5A, significant fluorescence emission was observed only when Fe_4_P_2_W_18_, TA, and H_2_O_2_ were present simultaneously. In contrast, no obvious fluorescence was detected in the control groups (including Fe_4_P_2_W_18_ alone, TA alone, and H_2_O_2_ alone). This phenomenon strongly demonstrates that Fe_4_P_2_W_18_ effectively catalyzes the decomposition of H_2_O_2_ to generate highly reactive ·OH [33]. These ·OH subsequently react with TA to produce 2-hydroxyterephthalic acid (TAOH), which exhibits strong fluorescence characteristics. To further confirm this result, we performed electron paramagnetic resonance (EPR) measurements on the reaction system. As shown in Figure 5B, a distinct quartet signal was observed in the EPR spectrum, which is consistent with the standard spectrum of ·OH. These experimental results not only confirm that Fe_4_P_2_W_18_ generates ·OH during the catalytic decomposition of H_2_O_2_ but also provide an important experimental foundation for further in-depth investigation of the reaction mechanism of this catalytic system.

### 3.3. Optimization of Conditions

**Figure 6:**
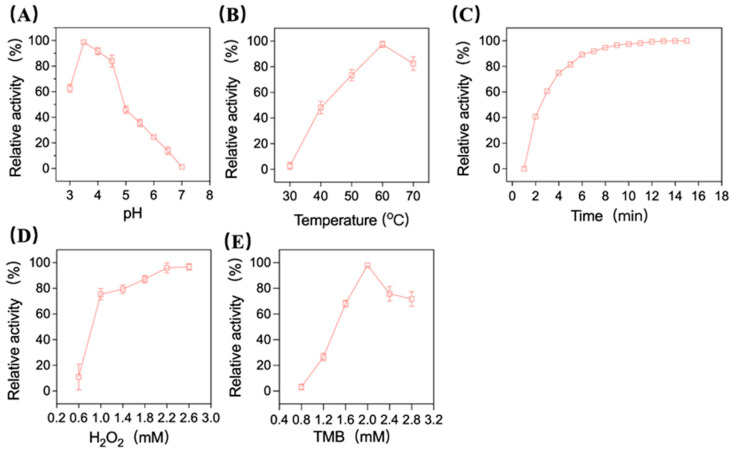
The optimization of the peroxidase-like activity of Fe_4_P_2_W_18_ based on the corresponding absorbance values: (**A**) pH, (**B**) temperature, (**C**) time, (**D**) H_2_O_2_ concentration, and (**E**) TMB concentration. The maximum point in each curve was set to 100%, and the error bars represent the standard deviation derived from three independent measurements.

The influence of various parameters on the peroxidase-like activity of Fe_4_P_2_W_18_ was systematically investigated to determine the optimal reaction conditions. As shown in Figure 6A, the absorbance increased with pH from 3.0 to 3.5, reaching a maximum at pH 3.5, and then decreased at higher pH values. This indicates that Fe_4_P_2_W_18_ exhibits optimal catalytic activity at pH 3.5, likely due to pH-induced changes in the structure and active sites of the nanozyme, which subsequently affect its interaction with substrates. The catalytic activity increased with temperature from 30 to 60 °C, peaking at 60 °C, followed by a slight decrease at higher temperatures (Figure 6B). Notably, Fe_4_P_2_W_18_ maintained high catalytic efficiency across the entire temperature range of 30–70 °C, demonstrating broad temperature adaptability. To ensure the stability of the analytes, a reaction temperature of 45 °C was selected for subsequent experiments. As depicted in Figure 6C, the catalytic reaction proceeded rapidly, reaching a plateau within 5 min. This fast catalytic kinetics meets the requirements for rapid detection. Optimization of TMB and H_2_O_2_ concentrations (Figure 6D,E) revealed that the optimal concentrations were 2 mM for TMB and 1 mM for H_2_O_2_, beyond which no significant increase in activity was observed. Collectively, the optimal reaction conditions were determined as follows: pH 3.5, temperature 45 °C, reaction time 5 min, TMB concentration 2 mM, and H_2_O_2_ concentration 1 mM. All optimization experiments were performed in triplicate to ensure data reliability.

### 3.4. Kinetic Analysis of Fe_4_P_2_W_18_ Nanozyme

**Figure 7:**
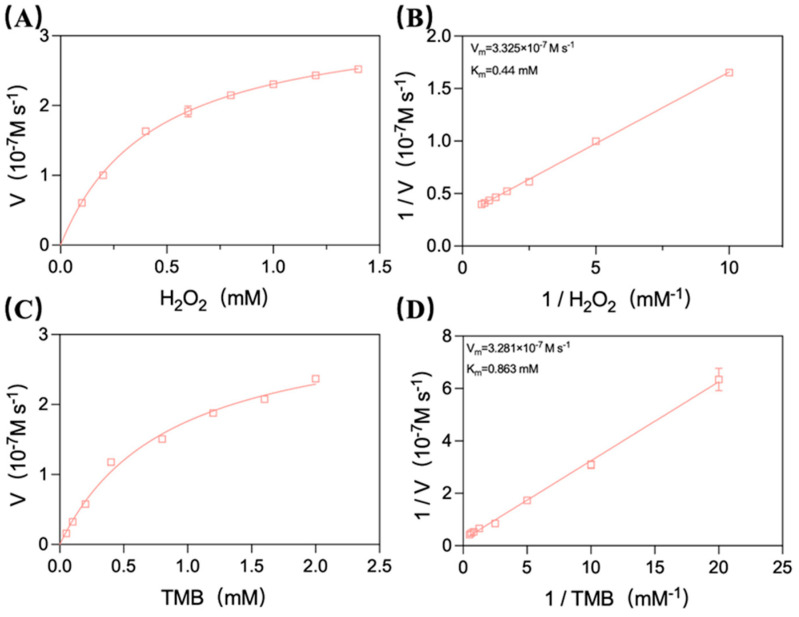
(**A**,**B**) Steady-state kinetic studies of Fe_4_P_2_W_18_ with H_2_O_2_ as the substrate. (**C**,**D**) Steady-state kinetic studies of Fe_4_P_2_W_18_ with TMB as the substrate. Error bars represent the standard deviation obtained from three parallel experiments.

Steady-state kinetic experiments were conducted to evaluate the catalytic performance of Fe_4_P_2_W_18_. The absorbance at 652 nm increased progressively with increasing concentrations of H_2_O_2_ and TMB, demonstrating the dependence of the catalytic reaction on substrate concentration. To quantitatively assess the catalytic efficiency, kinetic parameters were calculated by fitting the experimental data to the Michaelis–Menten model. The corresponding Lineweaver–Burk double-reciprocal plots (Figure 7A–D) yielded excellent linear fits, confirming that the catalytic behavior follows classical Michaelis–Menten kinetics [34]. For H_2_O_2_ as the substrate, the Michaelis constant (K_m_) and maximum reaction velocity (V_max_) were determined to be 0.44 mM and 3.325 × 10^−7^ M s^−1^, respectively. The relatively low K_m_ value indicates a high affinity of Fe_4_P_2_W_18_ toward H_2_O_2_. For TMB as the substrate, the K_m_ and V_max_ values were 0.863 mM and 3.281 × 10^−7^ M s^−1^, respectively. These results demonstrate that Fe_4_P_2_W_18_ exhibits favorable affinity and catalytic efficiency toward both substrates. When compared with natural horseradish peroxidase (HRP) (Table 2), the catalytic efficiency of Fe_4_P_2_W_18_ is comparable, further validating its potential as an effective peroxidase mimic for practical applications.

### 3.5. pKa-Driven and Enrichment-Synergistic Strategy for Biothiol Determination

**Figure 8:**
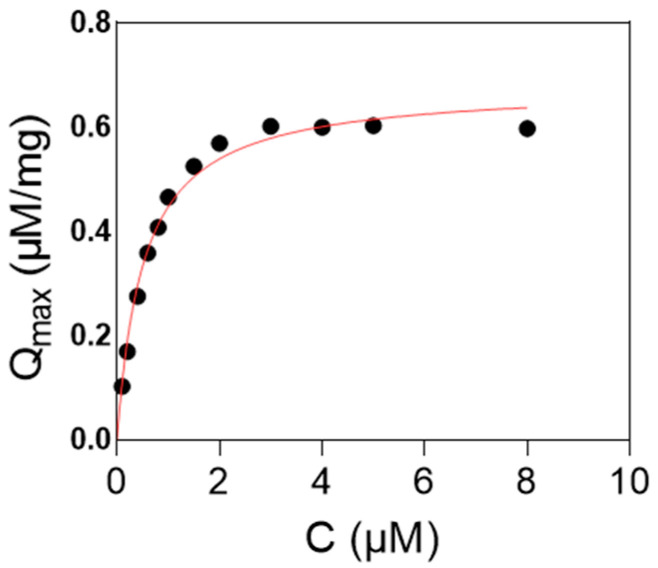
Simulation of the maximum adsorption capacity of Fe_4_P_2_W_18_ for biothiols based on the Langmuir adsorption model.

**Figure 9:**
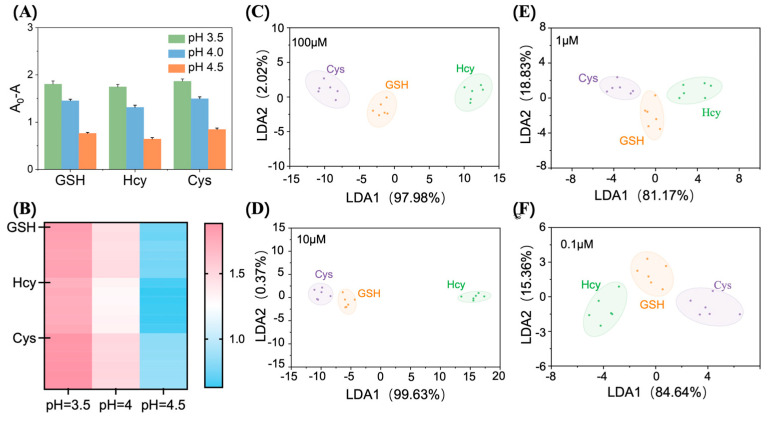
(**A**) Colorimetric response patterns (A_0_-A) of three thiols (100 μM); (**B**) Heat map of absorbance changes (A_0_-A) for three thiols (100 μM); (**C**–**F**) Two-dimensional linear discriminant analysis (LDA) plots of the responses to three thiols at different concentrations (100 μM, 10 μM, 1 μM, 0.1 μM) using the nanozyme-based sensor.

**Scheme 2:**
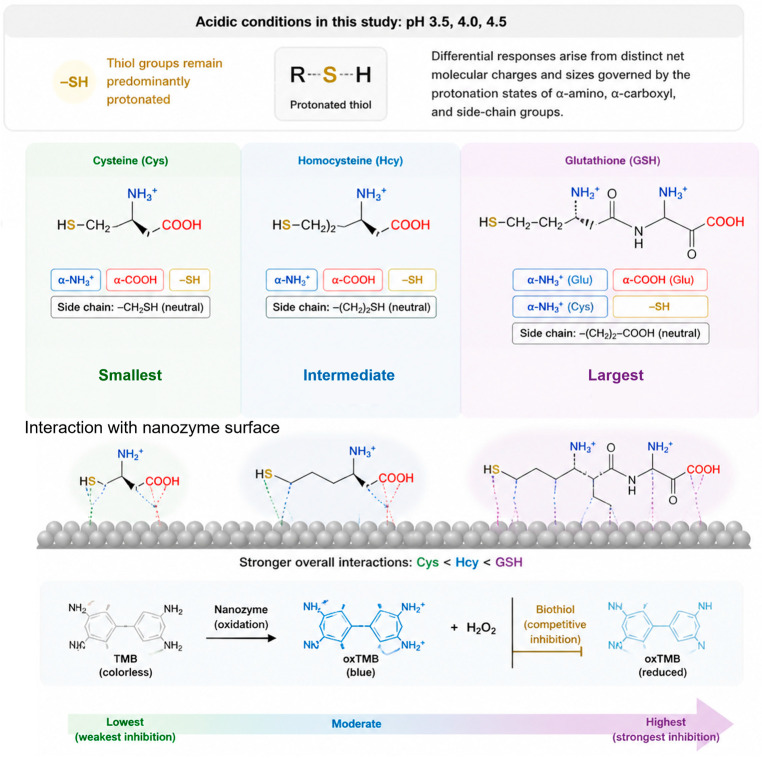
Schematic illustration of the detection mechanism for enrichment of biothiols (GSH, Cys, Hcy) onto the negatively charged Fe_4_P_2_W_18_ nanozyme surface. Differential responses arise from distinct net molecular charges and sizes governed by the protonation states of a-amino, a-carboxyl, and side-chain groups.

A colorimetric sensor array was constructed based on the pH-dependent peroxidase-like activity of Fe_4_P_2_W_18_, leveraging a pKa-driven and enrichment-synergistic strategy for biothiol discrimination. The polyoxometalate framework contains anionic clusters and multi-electron reduction centers, enabling the nanozyme to enrich biothiols via both surface-negative-charge-mediated electrostatic interactions and electron-transfer processes [35]. The maximum adsorption capacity (Q_max_) of Fe_4_P_2_W_18_ toward biothiols was determined to be approximately 0.68 μmol/mg, reflecting its efficient enrichment capability (Figure 8). Meanwhile, the thiol groups of GSH, Cys, and Hcy are weakly acidic and exhibit distinct pKa values (approximately 8.3 for Cys, 8.9 for Hcy, and 9.2 for GSH). Under the acidic pH conditions employed in this study (3.5, 4.0, and 4.5), these three biothiols exist predominantly in their protonated forms but with systematically varying protonation states due to their pKa differences. At these acidic pH values, the thiol groups remain predominantly protonated; the differential responses arise instead from the distinct net molecular charges and sizes governed by the protonation states of the α-amino, α-carboxyl, and side-chain groups. This variation leads to differential interactions with the nanozyme surface and distinct inhibition efficiencies toward the catalytic oxidation of TMB. By combining the enrichment capability of Fe_4_P_2_W_18_ with the pKa-driven differential responses of the three thiols across three finely tuned pH channels, the sensor array generates highly distinctive cross-reactive fingerprints (Scheme 2). The detection was performed at three different pH values (3.5, 4.0, and 4.5) to generate three sensing channels. Biothiols (GSH, Cys, and Hcy) inhibit the nanozyme-catalyzed TMB oxidation by scavenging reactive oxygen species (ROS), leading to a decrease in absorbance at 652 nm. The signal response (ΔA) was calculated as ΔA = A_0_ − A, where A_0_ and A represent the absorbance in the absence and presence of biothiols, respectively [36]. As shown in Figure 9A,B, the three biothiols exhibited distinct response patterns (bar chart and heat map) across the three pH channels, confirming the effectiveness of the sensor array for biothiol identification. Notably, at concentrations of 100, 10, 1, and 0.1 μM, all three biothiols were accurately discriminated without any misclassification (Figure 9C–F). The limit of detection (LOD) was calculated using the 3σ/slope method, where σ is the standard deviation of the blank signal (*n* = 3), and the slope was derived from the linear portion of the response–concentration curve. Even at an ultralow concentration of 0.1 μM, the sensor successfully distinguished GSH, Cys, and Hcy. This detection limit is significantly lower than the physiological concentrations of biothiols in serum, demonstrating the applicability of this array for real sample analysis. To rigorously validate the claimed 100% discrimination accuracy and exclude potential model overfitting, the LDA classification results were examined using confusion matrices and leave-one-out cross-validation (LOOCV). As shown in Table 3, at all four tested concentrations (100, 10, 1, and 0.1 μM), each of the three biothiols was correctly classified in all replicates, yielding a standard LDA accuracy of 100% (36/36). LOOCV was further performed by iteratively holding out one sample as the test set while training the model on the remaining 35 samples. The LOOCV-confirmed accuracy was also 100% at every concentration (Table 4), demonstrating that the perfect classification reflects genuine inter-class separability rather than overfitting to the training data.

### 3.6. Analytical Performance of the Sensor Array

The analytical performance of the Fe_4_P_2_W_18_ sensor array was systematically evaluated in terms of stability, reproducibility, repeatability, and selectivity. As summarized in Table 5, the nanozyme retained 98.4% of its initial catalytic activity after storage at room temperature for 7 days, demonstrating excellent stability. The batch-to-batch reproducibility was assessed using five independently synthesized batches, yielding relative standard deviations (RSD) of 2.1%, 1.9%, and 3.3% for GSH, Cys, and Hcy (10 μM each), respectively, all within the acceptable threshold of 5%. The repeatability, evaluated by ten successive measurements of GSH (10 μM) using a single sensor, gave an RSD of 1.8%, confirming the high precision of the measurement. Selectivity was evaluated by spiking each biothiol (10 μM) into a mixed interferent solution containing glucose, glycine, lysine, ascorbic acid, Na^+^, K^+^ (100 μM each), and BSA (100 μg/mL). The recoveries were 98.5%, 97.2%, and 96.8% for GSH, Cys, and Hcy, respectively, all within the 90–110% acceptance range, confirming the excellent anti-interference capability of the sensor array.

### 3.7. Quantitative Determination of GSH, Cys, and Hcy

**Figure 10:**
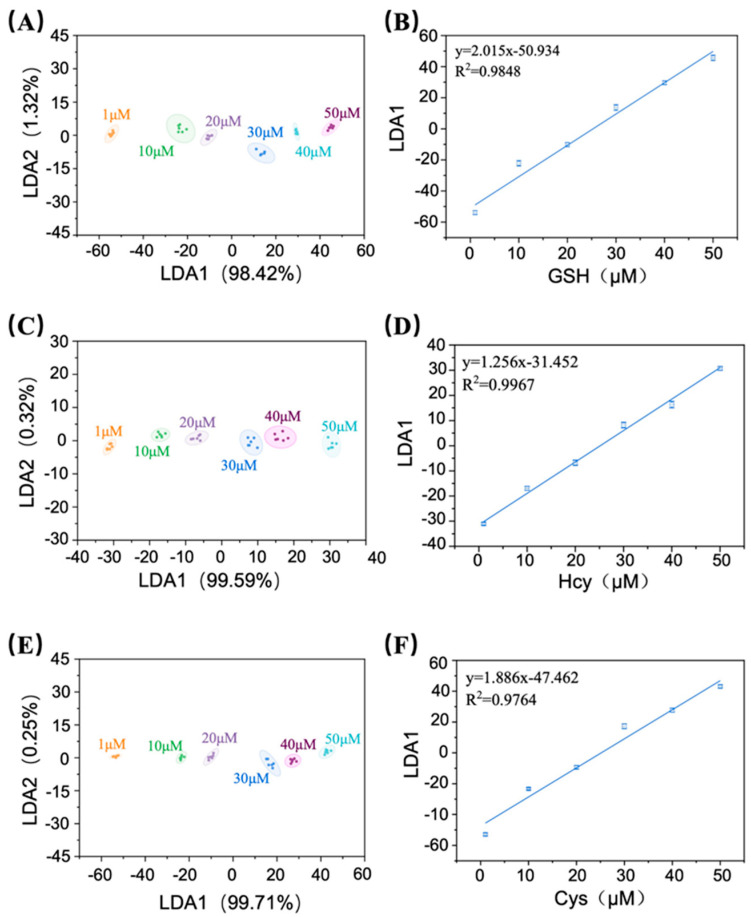
The Fe_4_P_2_W_18_ nanozyme sensor was used for quantitative detection of (**A**,**B**) GSH, (**C**,**D**) Hcy, and (**E**,**F**) Cys, respectively. From left to right, the two-dimensional linear discriminant analysis plots and the linear relationship plots between concentration and LDA1 are shown.

To evaluate the quantitative performance of the sensor array, biothiol samples at varying concentrations (1, 10, 20, 30, 40, and 50 μM) were systematically analyzed. As shown in Figure 10A, different concentrations of GSH were well separated in the two-dimensional LDA plot, with the first discriminant function (LDA1) accounting for 98.42% of the total variance—a value substantially higher than the acceptable threshold of 60%, indicating that LDA1 effectively captures the concentration-dependent variation [37]. A good linear relationship was established between LDA1 scores and GSH concentrations in the range of 1–50 μM (Figure 10B), with a regression equation of y = 0.032x − 0.518 (R^2^ = 0.994). This excellent linearity confirms the reliable quantitative detection of GSH using the sensor array. Similarly, quantitative discrimination and linear relationships were also achieved for Hcy (Figure 10C,D) and Cys (Figure 10E,F). For Hcy, the LDA1 contribution was 97.63% with a regression equation of y = 0.028x − 0.447 (R^2^ = 0.991); for Cys, the LDA1 contribution was 96.89% with a regression equation of y = 0.030x − 0.492 (R^2^ = 0.992). These results collectively demonstrate that the sensor array not only enables qualitative discrimination but also provides excellent quantitative capability for biothiol detection across a broad concentration range, making it suitable for practical applications where precise concentration determination is required. The predictive accuracy of the calibration models was further validated by leave-one-out cross-validation. The RMSEP values were determined to be 1.6, 1.8, and 2.0 μM for GSH, Cys, and Hcy, respectively, corresponding to 3.3%, 3.7%, and 4.1% of the calibration range (1–50 μM) (Table 6), confirming that the models are not overfitted and possess reliable quantitative predictive power.

### 3.8. Discrimination of Binary and Ternary Thiol Mixtures

**Figure 11:**
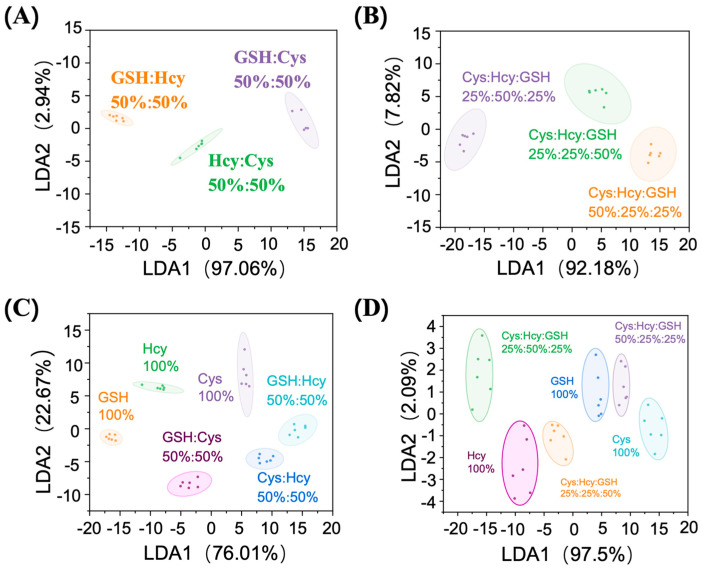
Two-dimensional linear discriminant analysis (LDA) plots of binary mixtures (**A**,**C**) and ternary mixtures (**B**,**D**) of different thiols.

To evaluate the capability of the sensor array for identifying thiol mixtures in complex scenarios, binary and ternary mixtures containing different molar ratios of GSH, Cys, and Hcy were analyzed. This investigation is particularly important because biological samples typically contain multiple biothiols simultaneously rather than a single species, and the ability to discriminate mixtures is essential for practical diagnostic applications [38]. Three binary mixtures (Cys/Hcy = 50:50, Hcy/GSH = 50:50, and Cys/GSH = 50:50) and three ternary mixtures (Cys/Hcy/GSH = 25:25:50, 50:25:25, and 25:50:25) were prepared, each with a total concentration of 100 μM. As shown in Figure 11A–D, all binary and ternary mixtures were clearly distinguished in the two-dimensional LDA plots, with distinct clustering centers showing no overlap. The cumulative variance explained by the first two discriminant functions exceeded 97% in all cases, confirming the high discrimination power of the sensor array [39]. The classification accuracy achieved for both binary and ternary mixtures was 100%, with no misclassification observed across all replicates, outperforming previously reported nanozyme array sensors (Table 7). These results demonstrate that the sensor array not only distinguishes individual biothiols but also accurately identifies their mixtures at various molar ratios, which is crucial for practical applications where biological samples typically contain multiple biothiols simultaneously. This capability, combined with the quantitative detection performance described above, positions the sensor array as a versatile tool for comprehensive biothiol analysis in complex biological matrices.

### 3.9. Real Sample Analysis

**Figure 12:**
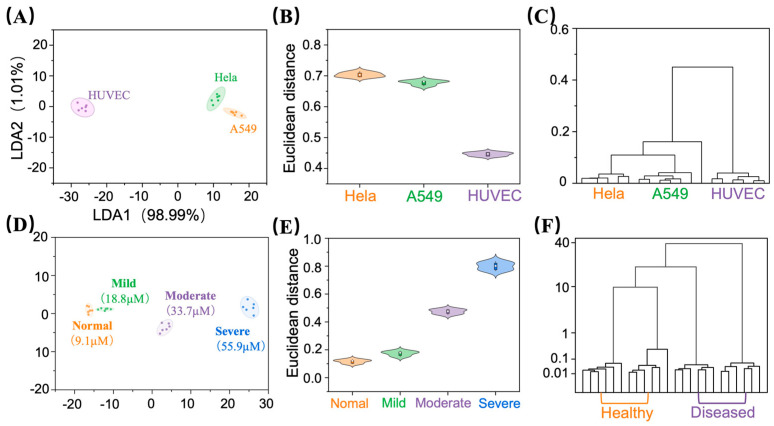
(**A**) Two-dimensional linear discriminant analysis (LDA) plot of three cell types based on different GSH concentrations using the sensor; (**B**) Violin plot of the recognition results for three cell types; (**C**) Hierarchical cluster analysis (HCA) dendrogram of three cell types at different GSH concentrations; (**D**) Two-dimensional LDA plot of serum samples with different Hcy concentrations; (**E**) Violin plot of the recognition results for four serum samples; (**F**) HCA dendrogram of serum samples with different Hcy concentrations.

The practical applicability of the sensor was validated by analyzing real biological samples, representing a critical step toward translation of this single-material array strategy from proof-of-concept to real-world diagnostic applications. Cancer cells often exhibit elevated intracellular GSH levels compared to normal cells due to altered glutathione metabolism [42]. Based on this established correlation, the sensor was employed to distinguish three cell types: HUVEC (normal), HeLa (cervical cancer), and A549 (lung cancer). As shown in Figure 12A, the three cell types were clearly separated in the LDA plot, with normal cells concentrated on the left and cancer cells on the right, demonstrating that the sensor array can effectively translate intracellular GSH level differences into distinct fingerprint responses. The violin plot (Figure 12B) further revealed significant differences between normal and cancer cells, as well as between the two cancer cell lines, highlighting the high sensitivity of the sensor to subtle variations in GSH levels. Hierarchical cluster analysis (HCA) confirmed that all cell samples were accurately classified without any misclassification (Figure 12C), achieving 100% accuracy. This successful cell typing underscores the key innovation of our strategy: by leveraging the pKa-driven differential responses of biothiols across three pH gradients, a single structurally well-defined Fe_4_P_2_W_18_ nanozyme can generate sufficiently orthogonal fingerprints to resolve complex biological samples, eliminating the need for multiple sensing materials.

The sensor was further applied to analyze Hcy levels in human serum samples, a clinically relevant application given the established link between elevated Hcy and cardiovascular disease [43]. Normal serum Hcy concentrations typically range from 5 to 15 μM, while patients with cardiovascular disease often exhibit significantly higher levels [44]. Four serum samples with different Hcy concentrations (9.1, 18.8, 33.7, and 55.9 μM) were analyzed. LDA revealed a clear distribution along the negative-to-positive axes, with low-concentration samples on the left and high-concentration samples on the right, without any overlap (Figure 12D). The violin plot (Figure 12E) showed increasing Euclidean distance with increasing Hcy concentration, clearly distinguishing normal from abnormal samples. HCA results further confirmed that the four serum samples were classified into four distinct groups, with normal and mild cases grouped as healthy (Figure 12F). These results validate the potential of the sensor for early diagnosis of cardiovascular disease. Importantly, the successful discrimination of serum samples with varying Hcy concentrations in a complex matrix such as human serum demonstrates the robustness of the sensor array against potential interferences, a direct benefit of the cross-reactive fingerprinting mechanism enabled by the single-material pH-gradient design. Although POMs may adsorb serum proteins, the cross-reactive fingerprinting strategy inherently tolerates such systematic background signals, as LDA extracts only the analyte-dependent differential responses across the three pH channels. Collectively, these real-sample validations highlight that our pKa-driven and enrichment-synergistic strategy not only simplifies sensor fabrication but also delivers reliable diagnostic information in clinically relevant contexts. To further validate the accuracy of the sensor array for quantitative analysis in complex biological matrices, spike-recovery experiments were performed. Human serum and HeLa cell lysate were spiked with GSH, Cys, and Hcy at a concentration of 10 μM. As shown in Table 8, the recoveries ranged from 91.0% to 108.0%, with RSD values between 2.9% and 4.8%, confirming the reliability of the sensor for accurate biothiol determination in real samples.

## 4. Conclusions

This work presents a conceptually distinct strategy for biothiol discrimination by synergistically regulating both nanozyme catalysis and thiol protonation states. Unlike conventional array sensors that rely on multi-material combinations or physical confinement, this approach utilizes a single polyoxometalate nanozyme (Fe_4_P_2_W_18_) with an atomically precise structure, which enables effective enrichment of biothiols through its negatively charged surface and multi-electron reduction centers. By leveraging the stepped catalytic response across three acidic pH gradients (3.5, 4.0, and 4.5), the system amplifies the inherent pKa-governed differences in biothiol molecular charge and structure, generating highly distinctive fingerprint responses. This single-material design eliminates the cumbersome synthesis and poor structural definition associated with multi-component arrays. At the same time, the well-defined molecular structure of Fe_4_P_2_W_18_ provides a reliable foundation for elucidating the catalytic and sensing mechanisms. The resulting sensor achieves 100% discrimination accuracy for GSH, Cys, and Hcy within 5 min, with successful application to cell typing and cardiovascular disease serum analysis, demonstrating a shift from multi-material complexity to single-material, mechanism-driven array sensing for early disease diagnosis.

## Figures and Tables

**Table 1:** Elemental Composition of Fe_4_P_2_W_18_ from XPS Survey and Narrow-Scan Analysis.

Element	Peak BE (eV)	FWHM (eV)	Atomic %	Assignment
**C 1s**	284.44	1.40	86.05	Adventitious carbon (charge reference)
**K 2p**	292.73	1.40	7.18	K^+^ counterion
**W 4f**	35.16 (W 4f_7/2_)	1.15	3.56	W^6+^ in POM framework
**Fe 2p**	711.00 (Fe 2p_3/2_)	3.09	1.88	Fe^3+^ in POM framework
**P 2p**	133.54	1.64	1.32	P^5+^ in POM framework

**Table 2:** The maximum reaction velocity (Vmax) and Michaelis−Menten constants (Km) of Fe_4_P_2_W_18_ nanozymes.

Nanozyme	Substrate	Vmax (×10^−8^ M·s^−1^)	Km (mM)
Fe_4_P_2_W_18_	H_2_O_2_	33.25	0.44
Natural HRP	H_2_O_2_	8.71	3.70

**Table 3:** Confusion Matrix of LDA Classification for GSH, Cys, and Hcy Differentiation.

Concentration (μM)	Actual Class	Predicted as GSH	Predicted as Cys	Predicted as Hcy	Total	Accuracy
**100**	GSH	3	0	0	3	100%
**100**	Cys	0	3	0	3	100%
**100**	Hcy	0	0	3	3	100%
**100**	Subtotal				9	100%
**10**	GSH	3	0	0	3	100%
**10**	Cys	0	3	0	3	100%
**10**	Hcy	0	0	3	3	100%
**10**	Subtotal				9	100%
**1**	GSH	3	0	0	3	100%
**1**	Cys	0	3	0	3	100%
**1**	Hcy	0	0	3	3	100%
**1**	Subtotal				9	100%
**0.1**	GSH	3	0	0	3	100%
**0.1**	Cys	0	3	0	3	100%
**0.1**	Hcy	0	0	3	3	100%
**0.1**	Subtotal				9	100%
**Overall**	—	—	—	—	36	100%

**Table 4:** Confusion Matrix and Cross-Validation Results for GSH, Cys, and Hcy Discrimination.

Concentration (μM)	Standard LDA Accuracy	LOOCV Accuracy
**100**	100% (9/9)	100% (9/9)
**10**	100% (9/9)	100% (9/9)
**1**	100% (9/9)	100% (9/9)
**0.1**	100% (9/9)	100% (9/9)
**Overall**	100% (36/36)	100% (36/36)

**Table 5:** Analytical Performance of the Fe_4_P_2_W_18_-Based Colorimetric Sensor Array.

Performance Metric	Condition	Result	Acceptance Criterion
**Stability**	Room temperature, 7 days	98.4% activity retained	≥90%
**Reproducibility (RSD)**	5 batches, GSH 10 μM	2.1%	≤5%
	5 batches, Cys 10 μM	1.9%	
	5 batches, Hcy 10 μM	3.3%	
**Repeatability (RSD)**	10 measurements, GSH 10 μM	1.8%	≤3%
**Selectivity**	GSH 10 μM in mixture	98.5%	90–110%
	Cys 10 μM in mixture	97.2%	
	Hcy 10 μM in mixture	96.8%	

**Table 6:** LOOCV results for the LDA1–Concentration Calibration Models of Individual Biothiols.

Analyte	TSS	RSS	RMSEP (LDA1)	RMSEP (μM)	RMSEP (%)
**GSH**	1.7415	0.0104	0.051	1.6	3.3
**Cys**	1.5308	0.0122	0.055	1.8	3.7
**Hcy**	1.3336	0.0120	0.055	2.0	4.1

**Table 7:** Comparison of detection limit, accuracy rate, and strategy with recently reported sensors for biothiol measurement.

Nanozyme	Detection Limit (GSH)	Detection Limit (Cys)	Accuracy Rate	Strategy	Ref.
Fe_4_P_2_W_18_	0.1 μM	0.1 μM	100%	Single-material pH-driven array	This work
CoFe_2_O_4_@CoFeCN	1.5 μM	1.5 μM	92%	5 × 5 multi-substrate array	[16]
MoS_2_@Pd	0.2 μM	0.2 μM	96%	Oxidase-like three-channel	[40]
Os/Ag NPs	0.2 μM	0.22 μM	<100%	Single-channel	[19]
M-CDs	5 nM	5 nM	<100%	Dual-material array	[15]
GSH-AuNCs	N/A	0.08 μM	<100%	Single-channel ligand optimization	[41]

**Table 8:** Spike-Recovery Results for Biothiols in Human Serum and HeLa Cell Lysate.

Sample Matrixcc	Analyte	Spiked (μM)	Measured (μM)	Recovery (%)	RSD (%)
**Human serum**	GSH	10	10.7	107.0	3.5
**Human serum**	Cys	10	9.1	91.0	4.2
**Human serum**	Hcy	10	10.3	103.0	2.9
**HeLa lysate**	GSH	10	9.4	94.0	4.8
**HeLa lysate**	Cys	10	10.8	108.0	3.6
**HeLa lysate**	Hcy	10	9.7	97.0	3.3

## Data Availability

The data supporting this article are available in the article and in its online SI.
